# Hexokinase 2 drives glycogen accumulation in equine endometrium at day 12 of diestrus and pregnancy

**DOI:** 10.1186/s12958-016-0223-4

**Published:** 2017-01-05

**Authors:** Sarah A. Bramer, Alysson Macedo, Claudia Klein

**Affiliations:** Department of Veterinary and Clinical Diagnostic Sciences, University of Calgary, Faculty of Veterinary Medicine, 3280 Hospital Dr NW, Calgary, AB T2N 4Z6 Canada

**Keywords:** Equine, Endometrium, Glycogen, Hexokinase 2

## Abstract

**Background:**

Secretion of histotroph during the prolonged pre-implantation phase in mares is crucial to pregnancy maintenance, manifested as increased embryonic loss in mares with age-related endometrial degeneration. Glycogen content of uterine histotroph is higher during the progesterone-dominated phase of the estrous cycle in mares, but regulatory mechanisms are not well understood.

**Methods:**

mRNA expression of glycogen-metabolizing enzymes (HK1, HK2, GSK3B, GYS1, PEPCK, PKM, PYGM) in endometrial samples were compared among mares in anestrus, estrus, and at Day 12 of diestrus and pregnancy. In addition, hexokinase 2 (HK2) activity was assessed using a colorimetric assay.

**Results:**

HK2 was the key regulator of glycogen accumulation during diestrus and pregnancy; hexokinase transcript abundance and enzyme activity were significantly higher during diestrus and pregnancy than estrus and anestrus. In addition, despite similar relative transcript abundance, hexokinase activity was significantly greater in the pregnant versus diestrous endometrium. Therefore, we inferred there was regulation of hexokinase activity through phosphorylation, in addition to its regulation at the transcriptional level during early pregnancy. Based on immunohistochemistry, HK2 was localized primarily in luminal and glandular epithelial cells, with weaker staining in stromal cells.

**Conclusion:**

Among glycogen metabolizing enzymes identified, expression of HK2 was significantly greater during the progesterone-dominated phase of the cycle.

## Background

There are two forms of nutrition for the developing equine conceptus. Histotroph, also known as “uterine milk”, is derived from the uterine glands and is the primary source of nourishment prior to placental development [2]. The importance of uterine secretions in early embryo development has been emphasized as far back as the third century B.C. by Aristoteles (cited in [11]). At approximately Day 40, implantation occurs and placentation commences, marking the beginning of hemotrophic nutrition [1]. For the remainder of gestation, both mechanisms of nutrition are integral to fetal development [1]. Regardless, given the prolonged pre-implantation period in the mare, adequate histotroph is of particular importance to maintenance of pregnancy. The majority of pregnancy loss in horses occurs before Day 45 [21]; although the underlying reasons are multifaceted, inadequate secretion of uterine histotroph due to age-related endometrial degeneration likely contributes to increased early embryonic loss in older mares [15].

Glycogen, a storage form of glucose molecules, is one of the endometrial secretions that comprise histotroph and contribute to a successful pregnancy in a variety of species, including mink and human [6, 19]. In the equine endometrium, glycogen content is highest during diestrus, lower during estrus, but almost absent during anestrus. This cyclic variation in glycogen content is likely controlled by variations in progesterone concentrations, characteristic of various phases of the estrous cycle [9, 13]. In muscle, the process of glycogen synthesis, glycogenesis, begins with conversion of glucose into glucose-6-phosphate by hexokinase (primarily Hexokinase 1 and 2; HK1 and HK2). Glucose-6-phosphate is isomerized to glucose-1-phosphate and is then converted into UDP-glucose. Through glycogen synthase (GYS1), the glucose of UDP-glucose is transferred to the non-reducing end of a glycogen molecule [8]. The activity of GYS1 is regulated by glycogen synthase kinase 3 beta (GSK3B); this enzyme phosphorylates (and thereby inactivates) GYS1. In human endometrium, activity of GSK3B is regulated by progesterone [23]. Glycogen degradation, glycogenolysis, is catalyzed by glycogen phosphorylase (PYGM), releasing glucose-1-phosphate, that can be converted into glucose-6-phosphate, that can enter the glycolytic pathway [6] or be hydrolyzed by glucose 6-phosphatase (G6PC3) to form glucose.

Glycogen metabolism in human endometrium has been studied extensively [19, 20, 23]. More recently, uterine glycogen metabolism has been investigated in mink [6, 22]. However, the presence and expression of glycogen-metabolizing enzymes in the equine endometrium have not been as well characterized. Objectives of the present study were to describe gene and protein expression, as well as enzyme activity of glycogen metabolizing enzymes (GSK3B, GYS1, PYGM, HK1, HK2, and PKM) in the endometrium during estrus, diestrus, pregnancy, and anestrus. To the best of our knowledge, no study demonstrating distribution and activity of glycogen metabolizing enzymes in equine endometrium during the estrous cycle and early pregnancy has been published. We hypothesized that expression of one or more glycogen metabolizing enzyme in the equine endometrium is higher during the progesterone-dominated phase of the estrous cycle than estrus or anestrus.

## Methods

### Mares and tissue collection

All procedures were approved by the University of Calgary’s Animal Care Committee (AC13-0068 and AC16-0002). Prior to tissue collection, all mares underwent a breeding soundness examination to assess reproductive soundness. Only healthy mares less than 14 years of age with no detectable abnormalities of the reproductive tract were used. Endometrial tissue samples were collected from estrous (*n* = 10), diestrous (*n* = 8), pregnant (*n* = 8), and anestrous (*n* = 6) mares. Reproductive status was determined via transrectal ultrasonography. Estrous samples were obtained when a 35-mm follicle and pronounced uterine edema was present. Diestrous samples and samples from pregnant mares were obtained 12 d post-ovulation, as described [17]. Anestrous samples were collected in the winter when mares were reproductively quiescent (confirmed through absence of ovarian follicles > 10 mm). Endometrial biopsies were collected transcervically using an alligator-type uterine biopsy forceps. Following collection, tissue samples were divided in half and were either preserved in 10% formalin for 24 h and stored in 70% ethanol until embedding in paraffin or snap-frozen in liquid nitrogen and stored at −80°C until further processing. Presence of age-related degenerative changes such as periglandular fibrosis was ruled out through light microscopic examination. All samples were classified as either category I or IIb according to the grading system introduced by Kenney [15]. Tissue samples were collected from 25 mares. Seven mares were used twice, and overall, 32 samples were collected.

### Isolation of RNA

Total RNA was isolated from 75–100 mg of endometrial tissue using TRIzol reagent (Thermo Fisher Scientific, Ottowa, ON, Canada) according to the manufacturer’s instruction. The RNA (~50 μg) was adjusted to total volume of 100 μL and precipitated using equal volumes of isopropanol and 1/10 volume of 3M sodium acetate pH 5.2. Following centrifugation and washing of the resulting RNA pellet using 75% ethanol, RNA was re-suspended in water and RNA concentration was measured using a NanoDrop (Thermo Fisher Scientific); samples with a 260/280 ratio close to 1.95, and a 260/230 ratio close to 2.0 were used for analysis.

### Real-Time RT-PCR

RNA samples (1 μg) were treated with DNase I, Amplification Grade (Thermo Fisher Scientific) for 15 min at room temperature, followed by heat inactivation with EDTA for 10 min at 65°C, and then reverse transcribed using a High-Capacity cDNA Reverse Transcription Kit (Thermo Fisher Scientific).

For each endometrial sample, the mRNA expression of seven transcripts, glyceraldehyde-3-phosphate dehydrogenase (*GAPDH*), glycogen synthase kinase 3 beta (*GSK3B*), glycogen synthase 1 (*GYS*1), glycogen phosphorylase (*PYGM*), hexokinase 1 (*HK1*), hexokinase 2 (*HK2*), and pyruvate kinase (*PKM*) were measured using real-time PCR. Primers specific for selected transcripts were designed using Primer-BLAST (National Center for Biotechnology Information; Table 1). Real-time PCR was performed using PowerUp SYBR Green Master Mix (Thermo Fisher Scientific) with the following conditions: one cycle of 10 min at 95°C; 40 cycles of 15 s at 95°C, 1 min at 60°C; followed by generation of a melt curve. Each reaction consisted of 5 μL of PowerUp SYBR Green Master Mix, 3.5 μL of ddH_2_O, 0.5 μL of 10 μM forward and reverse primers, and 0.5 μL of cDNA (total volume of 10 μL per well). All PCR was performed in duplicate. Negative controls consisted of water replacing cDNA and non-reverse transcribed RNA reactions. All reactions were automatically pipetted using the epMotion Automated Pipetting System (Eppendorf, Mississauga, ON, Canada). Primer efficiency was tested with 10-fold dilutions of pooled cDNA; it was confirmed that all primers had a PCR efficiency from 90 to 100%. Primer specificity was determined by melt-curve analysis yielding a single melting temperature for each product and Sanger sequencing to confirm product identity. The cycle number where the fluorescent signal crossed the threshold (Cq) was recorded. Replicate Cq were averaged and Real-time RT-PCR results were normalized to the housekeeping gene *GAPDH*, by subtracting the Cq for *GAPDH* from the Cq for each sample, to obtain delta Cq [18].

**Table 1:** Forward and reverse primer sequences used for Real-Time RT-PCR

Transcript	Forward primer sequence (5′ to 3′)	Reverse primer sequence (5′ to 3′)	Accession number
*GAPDH*	TTGTCAAGCTCATTTCCTGGTATG	GTTAGGGGGTCAAGTTGGGAC	NM_001163856.1
*GSK3B*	ACAGGCCACAAGAAGTCAGC	GATGGCAACCAGTTCTCCTGA	XM_005601948.2
*GYS1*	TGGAATCCCCACACTCCAGA	AGGCAATAGGCCAGGTTTCC	NM_001126125.2
*PYGM*	GAGATCAACCAGCGCTTCCT	GATCTCCGAGTGGATGCGAG	NM_001145253.2
*HK1*	GCTGAGGAAGCAAACGAACG	GTTCCTCCAAGATCCAGGGC	XM_005602426.2
*HK2*	CTCAGAGCGGCTCAAGACAA	GCACACCTCCTTGACGATGA	NM_001081776.1
*PKM*	AGAACTTGTGCGAGCCTCAA	CTGTCTGGTGATTCCGGGTC	NM_001143794.2

Glyceraldehyde-3-phosphate dehydrogenase (GAPDH); Glycogen synthase kinase 3 beta (GSK3B); Glycogen synthase 1 (GYS1); Glycogen phosphorylase (PYGM); Hexokinase 1 (HK1); Hexokinase 2 (HK2); Pyruvate kinase (PKM)

### Immunohistochemistry

Tissues were embedded in paraffin, sections were cut at 5 μM and mounted onto positively charged slides. Immunohistochemistry (IHC) was done with an automated IHC stainer, BOND-MAX (Leica, Richmond Hill, ON, Canada), as described [16]. A polyclonal rabbit anti-human hexokinase 2 antibody was used (Thermo Fisher Scientific; PA5-29326). As negative control, primary antibody was replaced with rabbit normal serum. The recombinant fragment used to generate the antibody (corresponding to amino acids 53 – 342 of human hexokinase 2) is 93% homologous to equine hexokinase 2, 73% homologous to equine hexokinase 1, and 58% homologous to equine glucokinase. The antibody cross-reacted with equine HK2, based on Western Blotting analysis (Thermo Fisher Scientific).

### Hexokinase Activity

Equine endometrial HK activity was measured using a Hexokinase Assay Kit (Abcam, Toronto, ON, Canada; ab136957). For each endometrial sample, 10 mg tissue was homogenized in 200 μL of ice cold Assay Buffer using a Dounce homogenizer. Samples were centrifuged for 5 min at 4°C at 15,300 g and the resulting supernatant was collected and stored on ice. For each tissue lysate, 1 μL of sample and 49 μL of Assay Buffer were added to each sample well, along with 50 μL of Colorimetric Reaction Mix, followed by 50 μL of NADH standards along with 50 μL of Colorimetric Reaction Mix. A positive control was prepared and 1 μL of positive control and 49 μL of Assay Buffer were added to the positive control well, along with 50 μL of Colorimetric Reaction Mix. Reaction mixtures were incubated at room temperature for 60 min (protected from light). At the end of the incubation period, optical density at 450 nm was determined using a microplate reader (Spectra Max i3x, Molecular Devices, Sunnyvale, CA, USA). In a preliminary trial, measuring optical density at 450 nm in a kinetic mode starting at 20 min of incubation, 60 min was confirmed to be the ideal incubation period. Hexokinase activity (expressed as nmol NADH generated) was calculated according to the manufacturer’s instructions (Fig. 3).

### Statistical analyses

Normality of data was confirmed with the D’agostino and Pearson normality test. Delta Cq values for estrous, diestrous, pregnant, and anestrous endometrium for *GSK3B*, *GYS1*, *PYGM*, *HK1*, *HK2*, and *PKM* were compared by one-way ANOVA. To determine differences in hexokinase activity in endometrial lysates, nmol NADH generated after 60 min of incubation were compared using one-way ANOVA. Statistical analyses and graphing of results were performed using GraphPad Prism 6 (GraphPad Software, Inc., La Jolla, CA) and *P* < 0.05 was considered significant.

## Results

### Real-time RT-PCR

**Fig. 1:**
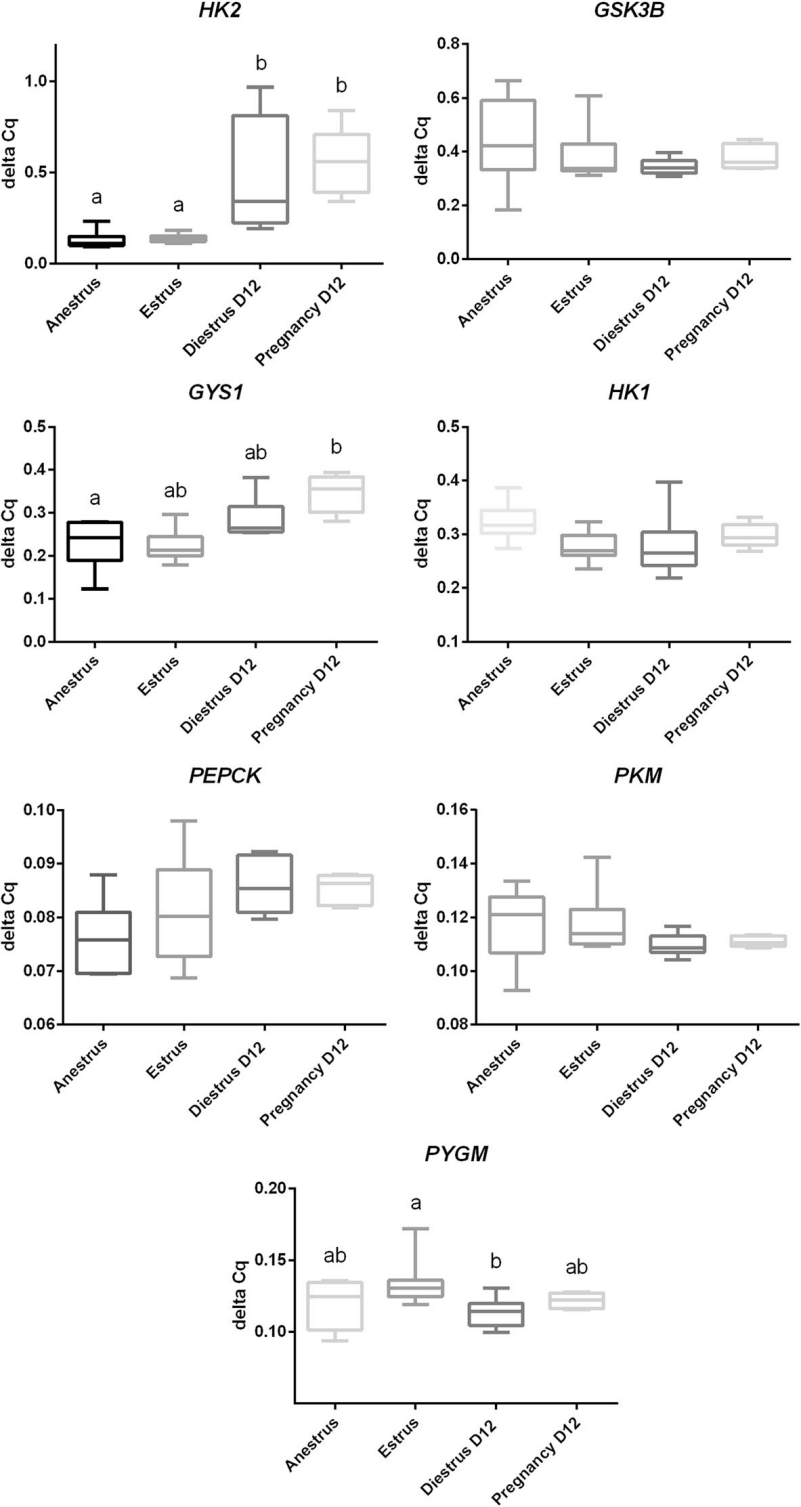
Relative transcript abundance of *HK2*, *GSK3B*, *GYS1*, *HK1*, *PEPCK*, *PKM*, and *PYGM* in equine endometrium during anestrus, estrus, Day 12 of diestrus and Day 12 of pregnancy. Relative expression levels are depicted as 1/deltaCq to account for the inverse relationship of deltaCq and relative transcript abundance. Means without a common superscript differed (*P* ≤ 0.05)

Relative transcript abundance of *HK2* was highest (*P* < 0.01) in samples collected during diestrus and pregnancy, with no difference between samples collected during anestrus versus estrus. Expression of *GYS1* in the endometrium was greater during pregnancy than anestrus (*P* < 0.01). Samples from estrous mares had a higher relative abundance of *PYGM* than samples collected from diestrous mares (*P* < 0.5). For *GSK3B*, *PYGM*, *HK1*, and *PKM*, there were no statistically significant differences in transcript abundance amongst the reproductive statuses examined. Note that results are displayed as 1 over delta Cq to account for the inverse relationship of delta Cq and relative mRNA abundance (Fig. 1).

### Immunohistochemical localization of endometrial HK2 expression

**Fig. 2:**
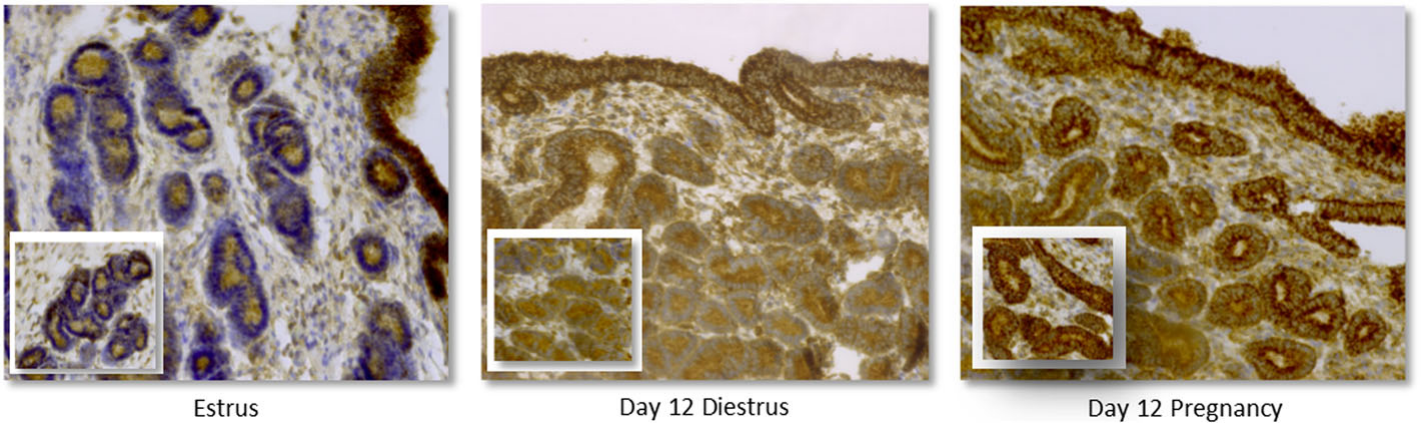
Immunohistochemical analysis of HK2 protein distribution in equine endometrium at estrus, and Day 12 of diestrus and pregnancy. Small inserted panels show glands located in the stratum compactum. No staining was observed in the negative control (not shown)

Based on immunohistochemistry, HK2 was localized primarily in luminal and glandular epithelial cells, with weaker staining in stromal cells. Staining intensity of luminal epithelial cells did not differ clearly between estrous, diestrous and pregnant samples, whereas staining intensity of glandular epithelial cells differed between the different stages examined. Staining intensity of glandular epithelial cells was strongest at Day 12 of pregnancy with slightly less pronounced staining observed at Day 12 of diestrus. Estrous samples displayed moderate staining intensity of glandular epithelial cells. No staining was observed in the negative control. Images were taken at 200× magnification (Fig. 2).

### Hexokinase Activity

**Fig. 3:**
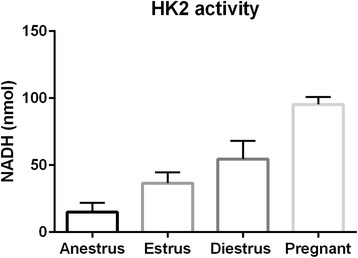
Hexokinase Activity (measured in nmol NADH produced by10 mg equine endometrium cell lysate after 60 min incubation time). Endometrial tissue samples were collected from mares during anestrus, estrus, diestrus, and pregnancy. All groups differ from each (*p* ≤ 0.05)

HK activity (as measured by nmol NADH produced during 60-min incubation) was higher in estrous than anestrous endometrial lysates (*P* < 0.05). Likewise, diestrous (*P* < 0.001) and pregnant (*P* < 0.0001) samples had higher hexokinase activity than anestrous samples. Compared to estrous samples, both diestrous (*P* < 0.5) and pregnant (*P* < 0.0001) samples were characterized by higher hexokinase activity. Pregnant samples displayed higher HK activity than diestrous samples (*P* < 0.001). Results are displayed in Fig. 3 as nmol NADH produced by 10 mg of endometrial lysate during 60-min incubation.

## Discussion

In this study, expression of glycogen metabolizing enzymes in equine endometrium during anestrus, estrus and 12 days post ovulation in cycling and pregnant mares was determined.. Herein, both mRNA and protein expression of HK 2, as well as HK activity were significantly greater during the progesterone-dominated phase of the estrous cycle. Hexokinase activity was assessed using a Hexokinase Assay Kit in which the conversion of glucose to glucose-6-phosphate by hexokinase is measured. Based on immunohistochemistry, HK 2 expression was most pronounced in glandular epithelial cells at Day 12 of pregnancy. Apart from HK 2, none of the other transcripts investigated (i.e. *GYS1*, *GSK3B*, *PYGM*, *HK1*, or *PKM*) had consistently higher expression during diestrus and pregnancy compared to estrus and/or anestrus.

The significance of histotroph in establishment and maintenance of pregnancy in the mare is well recognized and is manifested in increased embryonic loss in mares with endometrial fibrosis [15]. Various proteins are known components of uterine secretions in the mare, including uteroglobin [12], uterocalin [24], uteroferrin [25], and P19 [4]. Glycogen, through the release of glucose, is an integral constituent of histotroph; although accumulation of glycogen by the equine endometrium under the influence of progesterone has been described [9], expression of glycogen-metabolizing enzymes in the mare remains poorly defined. In human endometrium, expression of GSK3B is suggested to be regulated by progesterone, with higher expression of phosphorylated GSK3B (inactive) in secretory versus proliferative phases [23]. In mink, protein expression of HK1 and PYGM was greatest during estrus and diapause, whereas GYS protein expression was barely detectable after estrus [6]. Furthermore, G6PC3 gene expression was highest during diapause, suggesting that progesterone is glycogenolytic in the mink endometrium, as opposed to glycogenic in the human endometrium [6]. In the current study, HK2 was significantly upregulated during diestrus and pregnancy. Therefore, we inferred that transcriptional regulation of HK2 was involved in regulation of glycogen accumulation in the equine endometrium. Even though relative mRNA abundance of *HK2* did not differ between diestrus and pregnancy, HK2 activity was significantly higher in samples obtained from pregnant versus non-pregnant mares. Hexokinase activity can be regulated through phosphorylation, with phosphorylation resulting in increased activity [7]. Although phosphorylation status of HK2 was not evaluated in the present study, based on increased hexokinase activity in pregnant versus non-pregnant diestrous samples, we inferred that HK2 likely underwent differential phosphorylation during pregnancy. Glycogen Synthase 1 (GYS1), which catalyzes addition of glucose monomers to the growing glycogen molecule, had a trend for higher expression during the progesterone-dominated phase of the estrous cycle, although it was only significant when comparing samples obtained from anestrous mares with those obtained from pregnant mares.

A substantial source of economic loss in the equine breeding industry is attributed to pregnancy loss, which ranges from 7 to 17%, with greatest susceptibility prior to Day 60 [10]. More than 55% of pregnancy loss occurs by Day 39 and 75% by Day 49, with the risk of pregnancy loss decreasing as pregnancy progresses [3]. In early equine pregnancy, progesterone is essential for embryo survival; therefore, the absence of progesterone results in embryonic loss [10]. As mentioned previously, glycogenesis is mediated by progesterone in the human and equine endometrium [9, 14, 23]. Formation and accumulation of glycogen contribute to histotrophic nutrition, the sole form of early embryonic nutrition prior to establishment of haemotrophic nutrition. Therefore, decreased progesterone concentrations and/or a reduced response to progesterone could reduce the production of endometrial glycogen, increasing the probability of pregnancy loss. In one study [14] characterizing expression of secretory proteins, mucopolysaccharides, and glycogen in endometrial biopsies of mares with varying degrees of endometriosis, 32 of 48 biopsies had a cycle-asynchronous decrease in staining intensity for glycogen, implicating inadequate glycogen accumulation as a cause of increased embryonic loss in mares with degenerative endometrial changes. Given the frequent use of progesterone supplementation during early pregnancy in the mare [5], studies are warranted to determine whether exogenous progesterone during diestrus can further increase hexokinase activity, resulting in increased glycogen content of the endometrium.

## Conclusions

In this study, HK2 was the predominant enzyme controlling glycogen accumulation in the equine endometrium during diestrus and pregnancy. Follow-up studies should focus on comparing expression levels of HK2 between endometrial samples obtained from diestrous mares with varying Kenny and Doig scores (e.g. Grade 1 compared to Grades IIb or III) to determine whether HK2 expression is impaired with endometrial degeneration, and whether exogenous progesterone supplementation can increase hexokinase activity, and consequently, endometrial glycogen content.

## Abbreviations

GSK3B: Glycogen synthase kinase 3 beta
GYS1: Glycogen synthase 1
HK1: Hexokinase 1
HK2: Hexokinase 2
IHC: Immunohistochemistry
PEPCK: Phosphoenolpyruvate carboxykinase
PKM: Pyruvate kinase
PYGM: Glycogen phosphorylase.
